# Positive Microbiological Findings at the Site of Presumed Aseptic Revision Arthroplasty Surgery of the Hip and Knee Joint: Is a Surgical Revision Always Necessary?

**DOI:** 10.1155/2020/2162136

**Published:** 2020-05-09

**Authors:** Konstantinos Anagnostakos, Andreas Thiery, Christof Meyer, Ismail Sahan

**Affiliations:** Zentrum für Orthopädie und Unfallchirurgie, Städtisches Klinikum Saarbrücken, Saarbrücken, Germany

## Abstract

Little is known about patients that undergo presumed aseptic revision arthroplasty surgery of the hip and knee joint and having positive microbiological findings of the intraoperatively taken tissue samples. 228 “aseptic” operations were retrospectively analyzed from prospectively collected data with regard to the following parameters: demographic data; reasons for primary and revision surgery, respectively; time between primary and revision surgery; preoperative laboratory findings; microbiological and histopathological findings; type and length of systemic antibiotic therapy; clinical outcome; and follow-up. Identification of microorganisms was present in 8.8% of the cases (9.3% of the hip and 7.8% of the knee cases). Preoperatively, the median CRP value was 8.4 mg/l (normal values 0-5.0 mg/l) and the median WBC count 8,100 × 10^6^/l (normal values 3, 700‐10,100 × 10^6^/l). The most common identified organism was methicillin-resistant Staphylococcus epidermidis in 30%, followed by viridans streptococci in 15% of the cases. In 7 cases, the microbiological findings were interpreted as a contamination, and no antibiotic therapy was administered. In the other cases, a systemic antibiotic therapy was applied for a time period between 2 weeks and 3 months. 68.4% of the patients did not have any infectious complications at a median follow-up of 20 (3-42) months. The present study indicates that more than 2/3 of the cases with positive microbiological findings at the site of presumed aseptic revision arthroplasty surgery of the hip and knee joint can be successfully treated conservatively and they do not require any further surgical therapy.

## 1. Introduction

Periprosthetic joint infections (PJI) pose a rare but hazardous complication after total hip and knee arthroplasty. Treatment options mostly depend on the time of infection manifestation (early vs. low-grade vs. late infections). Although the exact time points of each infection type definition are not consistently accepted [1], orthopedic surgeons still agree that early periprosthetic infections might be successfully treated by debridement, antibiotics, irrigation, and retention of the prosthesis (DAIR) [2]. At the site of low-grade or late infections, treatment modalities always involve removal of the infected prosthesis, whereas some authors favor the one-stage [3, 4] and others the two-stage exchange arthroplasty [5–7].

In 1996, Tsukayama et al. firstly described a group of patients that underwent presumed aseptic revision arthroplasty surgery but had positive microbiological findings of the intraoperatively taken tissue samples [8]. Since then, only few studies have dealt with this topic [9–14], although clinical practice shows that orthopedic surgeons, who perform revision arthroplasty surgeries, are not infrequently confronted with this phenomenon. For these cases, the ideal further treatment remains unknown. It is unclear whether these patients require systemic antibiotic treatment and, if yes, for how long. Moreover, it is also unknown whether repeated prosthesis-retaining revisions or even a removal of the prosthesis should be planned.

The clinical relevance of such microbiological findings is still doubtful, and current literature data is scarce. Hence, the aim of the present retrospective study was to determine the incidence of positive microbiological findings at the site of presumed aseptic revision arthroplasty surgery of the hip and knee joint and evaluate whether these patients have abnormal serological indicators of PJI at the time of revision and what the outcome of these patients is.

## 2. Patients and Methods

Between January 2016 and December 2019, a total of 386 revision arthroplasty surgeries of the hip and knee joint were performed in our department. 158 surgeries were carried out due to septic reasons and were therefore excluded from the study.

The remaining 228 “aseptic” operations were retrospectively analyzed from prospectively collected data with regard to following parameters: demographic data (age, gender, and affected joint); reasons for primary and revision surgery, respectively; time between primary and revision surgery; preoperative laboratory findings (C-reactive protein (CRP); white blood cell (WBC) count); microbiological and histopathological findings; type and length of systemic antibiotic therapy (when applied); clinical outcome; and follow-up.

Before revision surgery, all patients were routinely given intravenous cefuroxime (or clindamycin for those with a history of allergy to penicillin or cephalosporins) before skin incision. During each surgery, samples of soft tissues from at least 3 different locations as well as of the joint fluid (when present) were taken and sent for further microbiological and histopathological examination. All samples were cultured in media for both aerobic and anaerobic microorganisms for at least 7 days. The histopathological findings were classified in accordance with the criteria proposed by Krenn et al. [15].

Informed consent was obtained from all patients.

## 3. Results

Among the 228 “aseptic” operations, 151 (66.2%) involved the hip and 77 (33.8%) the knee joint.

Identification of microorganisms was present in 20 cases (8.8%). There were 13 male and 7 female patients at a median age of 73.5 [38-86] years. The incidence of positive findings was 9.3% (14 out of 151 cases) for the hip and 7.8% for the knee joint (6 out of 77 cases).

In these 20 cases, the reasons for the revision surgery included prosthesis loosening in 15, instability in 3, and periprosthetic fractures in 2 cases. None of these patients had preoperatively any clinical signs of infection. The median time between the primary and the revision surgery was 86.5 [3-252] months (Table 1).

The preoperative median CRP value was 8.4 [<2.0-47.9] mg/l (normal values 0-5.0 mg/l). In the majority of the cases (14 out of 20), the CRP concentrations were normal or slightly elevated (<11 mg/l). Only in 3 cases the CRP values were beyond 30 mg/l. With regard to the WBC count, the median value was 8,100 [4, 900 − 14,900] × 10^6^/l (normal values 3, 700 − 10,100 × 10^6^/l). Only 3 patients had values greater than 11,000 × 10^6^/l (Table 2).

The most common identified organism was methicillin-resistant Staphylococcus epidermidis in 30% of the cases followed by viridans streptococci in 15% of the cases (Table 2). In 70% of the cases, the particular organism was detected in only one of the taken samples and in 30% of the cases in 2 or more (Table 2). In 30% of the cases, only 1 colony of the identified bacterium was present. The histopathological findings demonstrated an abrasion-induced periprosthetic membrane (type I) in 60% of the cases, a combined type (type III) in 30%, and a fibrous type (type IV) in 5% of the cases. A periprosthetic membrane of the infectious type (type II) could be detected in only one case (5%) (Table 2).

In 2 out of 20 cases, the surgeon made intraoperatively the decision to directly perform a two-stage protocol based on the macroscopic findings (Table 3). In the other 18 cases, the “aseptic” revision surgery was carried out as planned.

Postoperatively, all microbiological findings were discussed with our Microbiologic Institute. We decided to treat initially all cases conservatively and not to plan any further revision surgery. Taking into consideration the indication for the revision surgery, the intraoperative macroscopic evaluation of the particular situs, and the histopathological findings, the microbiological findings of 7 cases were interpreted as a contamination, and no antibiotic therapy was administered. In the other 13 cases, a systemic antibiotic therapy was applied for a time period between 2 weeks and 3 months (Table 3).

In the whole collective, 68.4% of the cases (13 out of 19) did not have any infectious complications at all at a median follow-up of 20 [3-42] months (Table 3). One patient passed away 6 weeks after the revision surgery due to cardiopulmonal decompensation and was therefore excluded from this evaluation.

In the group, where the findings were interpreted as contamination, 6 out of 7 patients had a completely uneventful course during follow-up. The remaining patient developed an arthrofibrosis of the operated knee joint within the first 8 postoperative weeks, which was regarded to be a sign of infection persistence. The patient then underwent revision surgery consisting of prosthesis explantation and implantation of an antibiotic-loaded spacer. The same pathogen organism could be identified as primary. This patient decided to retain his spacer and did not have any further surgeries (Table 3).

In the other group, where the patients were treated with systemic antibiotics, 5 out of 12 patients had to be treated for any infectious complications. Three of these patients underwent a two-stage procedure of the affected joint due to persistence of infection. One patient had revision arthroplasty surgery done after 10 days due to prolonged drainage; however, the microbiological findings were all negative. Nine days later, this patient developed a shoulder empyema with another bacterium. He underwent arthroscopic lavage and new antibiotic therapy for 4 weeks. The last patient had revision arthroplasty surgery done due to stem subsidence after 10 weeks. A different bacterium was identified than primarily, and the patient was treated again conservatively with antibiotics for 6 weeks. Among the two patients that underwent directly two-stage surgery, one decided to permanently retain the resection arthroplasty, and the other had successful prosthesis reimplantation after 7 weeks (Table 3).

The evaluation of the cases with regard to the infectious complications related to the number of positive/taken samples showed that 3 out of 11 cases (the two patients with direct two-stage protocol and the one who passed away are excluded) developed an infection when the organism was identified in only one sample and 3 out of 6 cases when positive in 2 or more.

## 4. Discussion

The aim of the present study was to determine the incidence of positive microbiological findings at the site of presumed aseptic revision arthroplasty surgery of the hip and knee joint and to evaluate whether these patients have abnormal serological indicators of PJI at the time of revision and what the outcome of these cases is. Our findings demonstrate a total incidence of 8.8% for both the hip and the knee joint. In the majority of the cases, the preoperative laboratory examination demonstrated normal values for both the CRP and the WBC count. Despite the positive microbiological findings, the histopathological examination could not confirm the presence of an infectious periprosthetic membrane in 65% of the cases. At a median follow-up of 20 months, more than 2/3 of our patients did not suffer from any infectious complications after being treated with systemic antibiotics or with no treatment at all.

The major challenge in revision arthroplasty surgery is the lack of a reliable and valid pre- and intraoperative diagnostic tool with 100% specificity and 100% sensitivity in the diagnosis or exclusion of a PJI [16]. Based on this problem, several criteria have been proposed by various societies, such as the Musculoskeletal Infection Society [17], the International Consensus Meeting on Periprosthetic Joint Infection [18], and the European Bone and Joint Infection Society [19], with none of them being universally accepted by now. Microbiological cultures are regarded to be the reference standard, but these are often false positive due to contamination or false negative due to changed growth characteristics of bacteria [13]. The use of molecular biological techniques, such as polymerase chain reaction (PCR), has been well investigated [13, 20, 21]; however, these techniques are very susceptible to contamination [13].

In the past years, some excellent scientific work has been increasingly made in this field; however, there still exists no tool or biomarker that is regarded to be the gold standard. Although a single abnormality in either the erythrocyte sedimentation rate or the CRP value has been reported to increase the likelihood of both infection and reoperation following revision arthroplasty [22], an elevation of the CRP values could be also attributed to other causes, such as cardiovascular, gastrointestinal, urologic, or respiratory problems or even unknown causes [23]. On the other side, normal CRP and WBC values cannot rule out a PJI [24]. Synovial biomarkers might play a role in the future but are currently not established in clinical setting. The analysis of antimicrobial peptides and proinflammatory cytokines might provide valuable information for the diagnosis of PJI [25]. Other authors described an increase in interleukins such as IL-1 and IL-6 in synovial fluid at the site of PJIs [26, 27]. The use of the synovial alpha-1-defensin [28, 29] and the synovial leucocyte esterase strip tests [28, 30] poses certainly an enhancement for the intraoperative diagnosis. However, disadvantages for both tests are well-known, such as the costs of the rapid lateral flow test of the former one and the possibility for blood within the synovial fluid to interfere with the color change of the urinalysis strip for the latter one [28]. Nuclear imaging techniques, such as leucocyte scintigraphy, have a sensitivity and specificity of 88% and 92%, respectively [31], but are not routinely performed depending on the particular surgical indication. Last but not least, the use of sonication has been demonstrated in several studies with promising results, whereas controversy still exists regarding the universal use of this technique [32], and this method does not help in the pre- and intraoperative setting.

The presence of a single positive culture still remains a matter of concern due to the difficulties in distinguishing true infection from contamination [14]. False-positive cultures may be seen in 13% of the cases [33]. The accurate interpretation of such a finding is of great clinical relevance, since the diagnosis of definitive PJI or only aseptic loosening with a contamination defines different therapeutic approaches.

Literature data is scarce about this topic. Based on the argument that patients suffering from an aseptic prosthesis loosening might contain a substantial number of cases with low-grade infections, which are missed with routine diagnostic tests, Moojen et al. conducted a prospective multicenter study to determine the incidence of these cases [13]. Among 176 patients with the preoperative diagnosis of aseptic loosening of their total hip arthroplasty, 4-13% of the cases were thought to be infected or suspected of infection based on the combined results of culture, histology, and broad-range 16S rRNA PCR with reverse line blot hybridization diagnostics. All patients but one had 1-stage revision with only 2 patients receiving antibiotic over a prolonged period. At 1-year follow-up, none of these patients had received any additional surgery. In a follow-up study by the same group, 173 of the 176 patients were followed for 6 to 9 years [10]. No significant differences in the number of rerevisions and the survival time of the particular implant were observed between the infection group and the aseptic loosening group. The authors concluded that a missed low-grade infection in patients diagnosed with aseptic loosening and receiving a revision total hip arthroplasty does not appear to influence the mid- to long-term prognosis.

Ribera et al. analyzed 89 cases with presumed aseptic loosening of a total hip or knee arthroplasty [14]. Depending on the evaluation of the tissue samples (TS) and the sonication fluid (SF), the patients were divided into 4 groups: group 1, consisting of ≥2 positive TS cultures; group 2, single positive TS culture and concordant SF; group 3, one positive or nonconcordant TS or SF-culture; and group 4: negative cultures. 12, 10, 38, and 29 patients were attributed to each group, respectively. The SF results correlated well with the TS ones in group 1; however, there was a great discrepancy in group 3 (positive TS culture in 32% versus positive SF culture in 74% of the cases). The authors concluded that the correct clinical interpretation of these cases is difficult.

In a retrospective multicenter study, Barrack et al. could determine unexpectedly positive intraoperative cultures in 41 out of 692 revision total knee arthroplasties (5.9%) [34]. 29 cases had a single positive intraoperative culture and were regarded to be false positive based on absence of any other evidence of infection. 24 of these patients received no treatment, and none of these 24 patients manifested any sign of infection at a mean follow-up of 46 months. The other 5 patients were treated with extended course of antibiotics. Twelve patients were thought to suffer from a periprosthetic infection, 11 of which were treated with a course of antibiotics. Two of these patients became reinfected within a year. The authors stated that a single positive intraoperative culture after revision total knee arthroplasty does not mandate further treatment in the absence of any other signs of infection.

Bereza et al. evaluated 37 cases with presumed aseptic hip or knee prosthesis loosening [9]. The average period between the first and second stage of the revision arthroplasty was 110 months. Elevated CRP concentrations were seen in 5 cases. Positive cultures of the sonicated fluid, intraoperative tissues, and joint liquid were observed in 29.7%, 18.9%, and 16.2% of the cases, respectively. The histopathological examination revealed evidence of infectious-type membrane in all cases of positive cultures and in 41.4% of the patients with negative cultures [9]. These results are in accordance with ours, with the presence of an infectious-type histopathological membrane in 35% of the cases.

Due to inhomogeneities in the study design, number of patients, microbiological methods used, and treatment options, a direct comparison of the available literature is almost impossible. However, the incidence of the positive microbiological findings in our study was similar to that reported in the literature [13, 34] so that we believe that this number might be regarded as “true,” independent of the department or originating country from the particular study. Of interest is also the fact that our collective did not solely include cases with presumed aseptic prosthesis loosening as in the other studies [13, 14, 34] but also instability and periprosthetic fracture cases. This points out the necessity of expanding the microbiological diagnostic measures onto other areas of periprosthetic revision surgery than only that of aseptic revisions.

In contrast to the statement of Barrack et al. that a single positive intraoperative culture after revision total knee arthroplasty does not mandate further treatment in the absence of any other signs of infection [34], approximately 30% of our cases with positive findings in a single sample developed an infection during follow-up, although some of them had been treated with systemic antibiotics. Moreover, one of the two patients that underwent directly a two-stage protocol due to the macroscopic findings at the revision surgery had positive findings in only one out of 5 examined samples. Both facts indicate that the identification of an organism in a sole sample does not surely exclude the presence of an infection or its future emergence, although the results of the present study do not allow for a valid statement about which patients truly require a specific therapy and which do not.

In our collective, a relative long median interval of approximately 7 years between the primary and the revision surgery was present. Similar numbers were provided in the study of Moojen et al. [13] with a median interval of 11 years and in the study of Bereza et al. with an average period of 11 years [9]. This evolves the question how the positive microbiological findings should be correctly described or interpreted in these cases. We disagree with the use of the terms “low-grade infection” or “subclinical infection,” because these patients did not have any clinical signs of infection, and under normal circumstances, any “low-grade” or “subclinical” infections should have expectedly caused any clinical complaints during such a period. Moreover, the universal use of this term for all cases is incorrect, because cases with a true contamination [33] would also be regarded to be “low-grade infections,” thus requiring further treatment, e.g., systemic antibiotic therapy. Although we cannot state for sure which term should be correctly used, we just want to emphasize that even the “correct” or “false” use of such terms might have medicolegal consequences.

Based on the aforementioned thoughts and facts, every orthopedic surgeon is confronted with the decision about the ideal treatment at the site of these cases. Theoretically, each “aseptic” revision surgery in this collective might be regarded to be a “septic” one-stage revision, since most of the single steps of the surgery (debridement and irrigation/pulsatile lavage) remain the same. In accordance with this principle, we decided not to routinely perform further revision surgery but to await the clinical course under systemic antibiotic therapy or at no therapy at all. We cannot predict what the outcome might have been if further surgical revisions would have been performed; however, the morbidity and the possible complications of additional surgeries should be taken into consideration before making such a decision.

A possible drawback of our study regards the incubation period of all samples for 7 days. Schäfer et al. reported that the detection rate via culture was 73.6% after 7 days and that especially late-detected organisms, such as Cutibacterium acnes, were isolated mainly after the first week, when the cultures were incubated for 14 days [35]. This implements for our study that the rate of positive cultures might have been higher if all samples had been incubated for 14 and not only 7 days. We acknowledge this problem and are planning to extend the incubation period to 14 days in the future. Furthermore, tissue samples were taken from at least 3 different locations and not 5 as frequently recommended in literature [36]. However, depending on the surgical indication, it is not always feasible or practicable to take tissue samples from 5 different locations, e.g., at the site of periprosthetic fractures or an isolated prosthetic component revision surgery. We usually aim to take samples from 5 completely different locations and not 5 samples from the same location. Nonetheless, this lower number of taken samples might have had an impact on the results; thus, the percentage of positive cultures might have been higher if at least 5 samples had been taken in each procedure and not only 3.

## 5. Conclusion

The present study could demonstrate that approximately one out of eleven revision arthroplasties of the hip and knee joint has positive microbiological findings of the intraoperatively taken samples. The usual preoperative laboratory examination consisting of CRP and WBC count is not helpful for confirming or excluding a possible PJI. A further revision surgery for treatment of the “infection” cannot be routinely recommended, since more than 2/3 of the patients remain free of infection after having systemic antibiotic therapy or no therapy at all.

## Figures and Tables

**Table 1:** Demographic data of the patients.

Patient	Gender	Age	Affected joint	Reason for primary surgery	Reason for revision surgery	Time between primary and revision surgery (months)
1	f	79	Hip	Periprosthetic fracture Vancouver type C after cemented hemiarthroplasty for femoral neck fracture	Stem loosening	9 after periprosthetic fracture/17 after cemented hemiarthroplasty
2	m	38	Hip	Femoral head necrosis	Recurrent dislocations	5
3	m	74	Knee	Degenerative osteoarthritis	Secondary PCL instability	5
4	m	86	Hip	Acetabular revision surgery	Cup loosening	52
5	f	62	Hip	Degenerative osteoarthritis	Cup loosening	128
6	m	48	Hip	Posttraumatric osteoarthritis	Cup loosening	44
7	m	78	Knee	Degenerative osteoarthritis	Tibial component loosening	132
8	m	57	Hip	Periprosthetic fracture Vancouver type B2 after cementless total hip arthroplasty	Stem subsidence	10
9	f	72	Hip	Aseptic THA revision	Cup loosening	85
10	f	52	Knee	Aseptic TKA revision	Tibial component loosening	70
11	m	62	Hip	Degenerative osteoarthritis	Stem loosening	108
12	m	60	Knee	Degenerative osteoarthritis	Mediolateral instability	100
13	m	78	Knee	Degenerative osteoarthritis	Femoral component loosening	92
14	m	82	Hip	Septic two-stage revision	Stem loosening	88
15	m	80	Knee	Septic two-stage revision	Tibial component loosening	10
16	m	73	Hip	Degenerative osteoarthritis	Cup loosening	252
17	f	83	Hip	Degenerative osteoarthritis	Periprosthetic fracture Vancouver type B2	192
18	f	81	Hip	Degenerative osteoarthritis	Cup loosening	132
19	m	68	Hip	Femoral neck fracture	Periprosthetic fracture Vancouver type B2	3
20	f	86	Hip	Degenerative osteoarthritis	Stem loosening	180

f: female; m: male; PCL: posterior cruciate ligament; THA: total hip arthroplasty; TKA: total knee arthroplasty.

**Table 2:** Laboratory, microbiological, and histopathological findings at the site of presumed aseptic revision arthroplasty surgery of the hip and knee joint.

Patient	Preop. CRP (mg/l)	Preop. WBC (×10^6^/l)	Microbiological findings	No. of positive/No. of taken samples	Histopathological findings
1	47.9	7,800	MRSE	1/3	Type III
2	8.5	10,400	MRSE	1/3	Type I
3	10.0	5,900	MRSE	2/3	Type I
4	5.5	14,900	MRSE	1/3	Type I
5	2.9	6,900	CNS^∗^	1/3	Type IV
6	6.1	8,400	Staphylococcus capitis Staphylococcus auricularis CNS	1/3	Type III
7	<2.0	6,800	Viridans streptococci^∗^	1/5	Type I
8	10.6	5,200	MRSE^∗^	1/1	Type I
9	<2.0	4,900	Gram-positive rods (coryneform)	1/3	Type I
10	10.1	11,400	Staphylococcus hominis^∗^	1/4	Type I
11	8.3	13,100	MSSE	1/3	Type III
12	4.8	10,800	Viridans streptococci^∗^	1/3	Type I
13	6.8	9,200	MRSE	3/3	Type I
14	32.0	11,000	Staphylococcus capitis^∗^	1/4	Type I
15	11.6	9,300	Staphylococcus hominis	2/3	Type III
16	42.2	7,200	Escherichia coli	1/3	Type III
17	12.6	7,700	Citrobacter koseri	2/3	Type II
18	4.2	5,300	Cutibacterium acnes	2/3	Type I
19	17.7	5,100	Gram-negative rods (no specification)	1/5	Type I
20	5.1	8,500	Streptococcus oralis	2/4	Type III

CRP: C-reactive protein; WBC: white blood cell; MRSE: methicillin-resistant Staphylococcus epidermidis; CNS: coagulase-negative staphylococci; MSSE: methicillin-susceptible Staphylococcus epidermidis; ^∗^only 1 colony.

**Table 3:** Therapy details and outcome.

Patient	Systemic antibiotic therapy	Infection persistence	Outcome	Follow-up (months)
1	Cefuroxime for 6 weeks	No	Intraop. decision for two-stage protocol, permanent Girdlestone	42
2	Linezolid for 2 weeks	Yes	Two-stage protocol after 1 year	40/28
3	None	Yes	Arthrofibrosis within 8 weeks, infection persistence; permanent spacer after 7 months	39
4	Ceftriaxone for 2 weeks	No	Revision surgery after 10 days due to prolonged drainage, negative microbiology, shoulder empyema after 9 days (E. cloacae), linezolid for 4 weeks	31
5	None	No	Uneventful course	30
6	Rifampicin+teicoplanin for 2 weeks	No	Uneventful course	29
7	Levofloxacin for 6 weeks	No	Intraop. decision for two-stage protocol, reimplantation after 7 weeks	28
8	None	No	Uneventful course	25
9	None	No	Uneventful course	23
10	None	No	Uneventful course	20
11	None	No	Uneventful course	18
12	Rifampicin+levofloxacin for 4 weeks	No	Uneventful course	17
13	Rifampicin+levofloxacin for 3 months	No	Uneventful course	15
14	None	No	Prosthesis dislocation after 1 week, open reduction	14
15	Rifampicin+teicoplanin for 4 weeks, followed by rifampicin+linezolid for 2 weeks	Yes	Two-stage protocol after 6 months	3
16	Rifampicin+levofloxacin for 6 weeks	Yes	Death due to cardiopulmonal decompensation after 6 weeks	n.r.
17	Meronem+ciprofloxacin for 6 weeks	Yes	Two-stage protocol after 2 weeks	3
18	Rifampicin+levofloxacin for 6 weeks	No	Uneventful course	6
19	Rifampicin+Meronem for 4 weeks	Yes	Revision due to stem subsidence after 10 weeks, identification of MSSE, conservative treatment	4
20	Rifampicin+levofloxacin for 6 weeks	No	Uneventful course	3

n.r.: not relevant.

## Data Availability

"The data used to support the findings of this study are available from the corresponding author upon request".
